# *Vibrio vulnificus* Phage PV94 Is Closely Related to Temperate Phages of *V. cholerae* and Other *Vibrio* Species

**DOI:** 10.1371/journal.pone.0094707

**Published:** 2014-04-14

**Authors:** Mark Pryshliak, Jens A. Hammerl, Jochen Reetz, Eckhard Strauch, Stefan Hertwig

**Affiliations:** Federal Institute for Risk Assessment (Bundesinstitut für Risikobewertung), Department of Biological Safety, Berlin, Germany; Free University of Berlin, Germany

## Abstract

**Background:**

*Vibrio vulnificus* is an important pathogen which can cause serious infections in humans. Yet, there is limited knowledge on its virulence factors and the question whether temperate phages might be involved in pathogenicity, as is the case with *V. cholerae*. Thus far, only two phage*s* (SSP002 and VvAW1) infecting *V. vulnificus* have been genetically characterized. These phages were isolated from the environment and are not related to *Vibrio cholerae* phages. The lack of information on temperate *V. vulnificus* phages prompted us to isolate those phages from lysogenic strains and to compare them with phages of other *Vibrio* species.

**Results:**

In this study the temperate phage PV94 was isolated from a *V. vulnificus* biotype 1 strain by mitomycin C induction. PV94 is a myovirus whose genome is a linear double-stranded DNA of 33,828 bp with 5′-protruding ends. Sequence analysis of PV94 revealed a modular organization of the genome. The left half of the genome comprising the immunity region and genes for the integrase, terminase and replication proteins shows similarites to *V. cholerae* kappa phages whereas the right half containing genes for structural proteins is closely related to a prophage residing in *V. furnissii* NCTC 11218.

**Conclusion:**

We present the first genomic sequence of a temperate phage isolated from a human *V. vulnificus* isolate. The sequence analysis of the PV94 genome demonstrates the wide distribution of closely related prophages in various *Vibrio* species. Moreover, the mosaicism of the PV94 genome indicates a high degree of horizontal genetic exchange within the genus *Vibrio*, by which *V. vulnificus* might acquire virulence-associated genes from other species.

## Introduction

*Vibrio vulnificus* is an estuarine bacterium which occurs in coastal waters worldwide [1]. The species is an important pathogen that can cause severe wound infections with lethal outcome [2]. In addition, *Vibrio vulnificus* is responsible for death cases caused by consumption of contaminated seafood [3]. In the Unites States it is a leading cause of seafood-related deaths [4]. Environmental factors, such as warm water and moderate salinity, are known to favor the multiplication of the pathogen. Hence, the effect of global warming on seawater temperatures has aroused concerns that *V. vulnificus* infections will increase in numbers [5], [6]. To date only limited information is available about the pathogenicity of this species and on the question what discriminates highly virulent strains from environmental strains with presumably lower virulence. Even though some virulence factors like e.g. capsular polysaccharide [7] and RTX toxins [8] have been identified, the full spectrum of factors has still to be elucidated. In *V. cholerae*, bacteriophages play a critical role in pathogenicity as cholera toxin is encoded by a temperate phage residing in the chromosome of toxigenic stains. In addition, K139-related phages which are widely distributed throughout different serogroups and biotypes of *V. cholerae* might be important for pathogenicity. The secreted virulence determinant gene *glo* (G protein-like ORF) of K139 lysogens has already been characterized [9]. In contrast to *V. cholerae*, little is known about *V. vulnificus* phages. Some studies describe the isolation of *V. vulnificus* phages from estuarine habitats [10], [11]. In oysters up to 10^5^ phages per g tissue have been found [10]. Based on morphological evidence, the phages belonged to the families *Myoviridae*, *Podoviridae* and *Siphoviridae*. They mainly lysed *V. vulnificus* strains, even though infection of *V. fluvialis* and *V. parahaemolyticus* by single phages has also been reported. Only two *V. vulnificus* phage genomes have been published until now [12], [13]. While the podovirus VvAW1 isolated from surface water in Hawai, USA, has been suggested to be temperate, the siphovirus SSP002 recovered from the coastel area of the Yellow Sea of South Korea may be virulent [12]. The VvAW1 38 kb genome revealed some similarities to non-*Vibrio* phages (e.g. *Thalassomonas* phage Ba3, *Pseudomonas* phage F116) whereas SSP002 is closely related to the *V. parahaemolyticus* phage vB_VpaS_MAR10 [14].

In this study we characterized the temperate *V. vulnificus* phage PV94 isolated from a clinical biotype 1 strain. PV94 is a myovirus whose genome shows a modular organization and mosaicism. The phage is closely related to *V. cholerae* K139-like phages and phages of other *Vibrio* species.

## Materials and Methods

### Isolation and purification of PV94 particles

To isolate PV94, the host strain *V. vulnificus* VN-094 (human isolate, strain collection of the BfR) was grown in Luria Bertani (LB) broth supplemented with 2% sodium chloride (w/v) to an optical density of 0.2–0.3 (A588). Induction of PV94 was performed by adding 0.25 µg/ml mitomycin C to the culture followed by further shaking for 6 to 18 h at 37°C until lysis occurred. High-titer phage lysates were obtained by preparing 500 ml cultures. Upon prophage induction, lysates were centrifuged for 30 min at 12,000×*g* and passed through 0.45 µm and 0.22 µm filters (Schleicher & Schüll, Dassel, Germany). Between the filtration steps, 10 mM MgCl_2_, 1 µg ml^−1^ DNase I, and RNase A (Roche, Mannheim, Germany) were applied to the lysates, which were incubated at 37°C for 2 h. Phage particles were concentrated by ultracentrifugation at 230,000×g for 2 h. Pellets were suspended in SM buffer and purified through discontinuous cesium chloride (CsCl) gradients (1.35 to 1.65 g ml^−1^), as previously described [15].

### Transmission electron microscopy (TEM)

The morphology of the phage was analysed by TEM using the negative staining procedure. Briefly, CsCl-purified phages were allowed to adsorb on pioloform-carbon-coated, 400-mesh copper grids (Plano GmbH, Wetzlar, Germany) for 10 min. Thereafter, the grids were fixed with a 2.5% aqueous glutaraldehyde (Taap Laboratories, Aldermaston, United Kingdom) solution for 1 min, and stained with 2% aqueous uranyl acetate (Merck, Darmstadt, Germany) solution for 1 min. The specimens were examined by TEM using a JEM-1010 (JEOL, Tokyo, Japan) at 80 kV accelerated voltage.

### Mass spectrometrical analysis of structural proteins

For the determination of PV94 structural proteins, CsCl-purified phages were disintegrated by boiling in SDS buffer for 10 min. Proteins were subjected to SDS-PAGE as described previously [16]. Bands of interest were excised and prepared for tryptic *in gel*-digests. The extracted peptides were dried, reconstituted and subjected to tandem matrix-assisted laser desorption ionization-time-of-flight mass spectrometry (MALDI-TOF-TOF MS/MS) analysis. Mass spectra were analyzed and interpreted using the Mascot software (Matrix Science Ltd., London, United Kingdom) followed by NCBInr database (NCBI) searches.

### Sequence determination and analysis of the PV94 genome

First sequence data of the phage were obtained by molecular cloning and sequencing of EcoRI and HindIII restriction fragments. For this purpose phage DNA was isolated from purified particles applying standard protocols [15]. Upon cleavage with EcoRI and HindIII (Thermo Scientific, Hudson, NA), restriction fragments were ligated to the corresponding sites of the vector pIV2 [17] and introduced into the *E. coli* strain Genehogs (Life Technologies, Darmstadt, Germany) by electroporation. Transformants harboring restriction fragments of the phage were identified by blue/white selection on LB agar containing 100 µg ml^−1^ neomycin. The inserts of recombinant plasmids were sequenced with vector primers and primers deduced from determined sequences. Gaps between the respective PV94 restriction fragments were closed by primer walking using phage DNA as template.

Sequence analyses and alignments were carried out using the Accelrys DS Gene software package of Accelrys Inc. (USA). ORF Finder of the NCBI database was used to predict putative open reading frames (ORFs) on the genome of PV94. Similarity and identity values nucleotide and amino acid sequences were determined using the algorithms of BLAST search (NCBI) [18]. Putative Rho-independent transcription terminators were identified using TransTerm [19], [20]. The complete nucleotide sequence of PV94 was compared with the genomes of the phages K139 (Accession number: AF125163) [21], kappa (AB374228) and VD1 (JF974301) and with a prophage residing in chromosome 1 of the *V. furnissii* strain NCTC 11218 (NC_016602, chr 1 positions ∼2,595,000 to ∼2,625,000) [22].

### Analysis of the cohesive ends

Cohesive ends of PV94 were identified by digestion of phage DNA with AdeI, BglII and SspI (Thermo Scientific, Hudson, NA) followed by an incubation of the digests at 70°C for 10 min. Thereafter, the obtained restriction patterns were compared to unheated control digests. In a further experiment, PV94 DNA that had been treated before with T4-ligase was digested and analysed. To determine the exact sequence of *cos*, primers deduced from a region close to the cohesive ends were used for direct sequencing of T4-treated and untreated phage DNA.

## Results and Discussion

**Figure 1:**
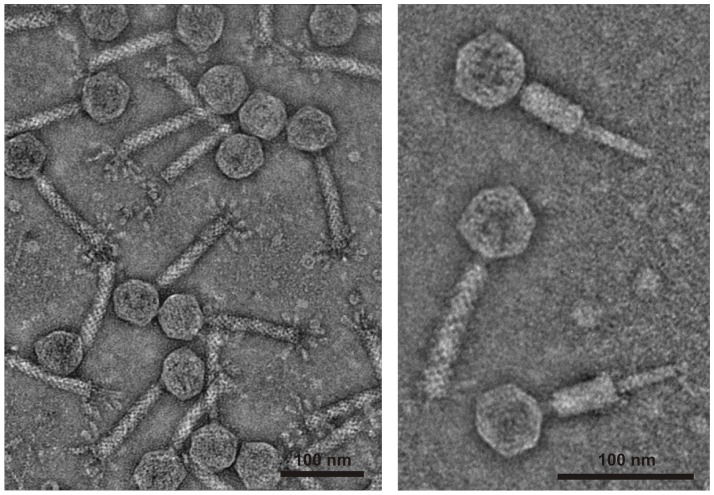
Morphology of phage PV94. Transmission electron micrographs of PV94 particles.

Phage PV94 was isolated from the *V. vulnificus* biotype 1 strain VN-094 by mitomycin C induction. Similar to other *Vibrio* strains, a very low concentration of the agent was optimal for phage release. PV94 particles revealed typical characteristics of a myovirus, an isometric head (56 to 58 nm in diameter) and a contractile tail (approximately 114 nm in length and 18 nm in width). At the end of the tail, a bundle of up to eight thick tail fiber-like structures was visible (Fig. 1). Although the virions appeared intact, no indicator strain suitable for phage propagation could be found. For this study 58 *V. vulnificus* and 25 *V. cholerae* strains, half of each clinical and environmental isolates, were tested by spot assays. To obtain high numbers of phages sufficient for purification by CsCl step gradients, large volumes of the host strain were induced with mitomycin C (see Materials and Methods). By this procedure a large amount of phage was isolated from which linear, double-stranded DNA was extracted.

**Figure 2:**
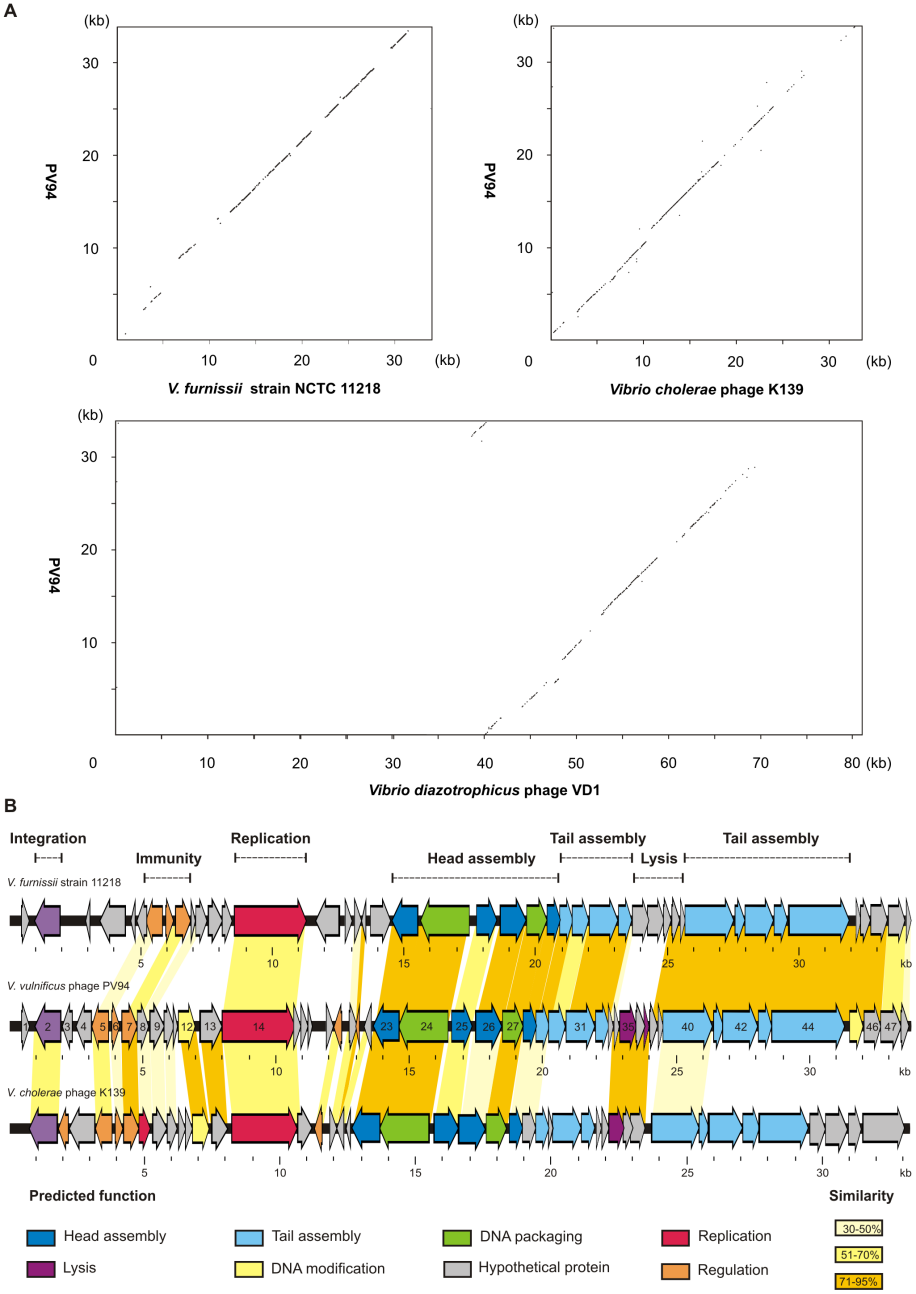
Relationship of PV94 to other *Vibrio* phages. (A) Dot plots of the genome of PV94 with genomes of *V. cholerae* phage K139, *V. diazotrophicus* phage VD1 and a cryptic prophage of the *V. furnissii* strain NCTC 11218. The axes of abscissas and ordinates show the coordinates of the respective phage genomes. (B) Genome organization of the phages PV94, K139 and the prophage of *V. furnissii* strain NCTC 11218. Colours indicate the predicted functions of gene products. Related genes of the phages are connected by coloured shading.

The PV94 genome consists of 33,828 bp with a G+C content of 48.2%, slightly higher than those of its host. We identified 48 possible Open Reading Frames (ORFs) of which 39 are located on one strand and the remaining nine on the second and 21 putative transcription terminators (Table S1 and Table S2). The strongest overall DNA homologies were detected to the *V. cholerae* phage K139 and related phages belonging to the kappa family, to the *V. diazotrophicus* phage VD1 and to a prophage residing in the genome of *V. furnissii* strain NCTC 11218 (Fig. 2A). In addition, PV94 shows some relationship to the *Aeromonas media* phage ΦO18P [23]. For 29 of the predicted PV94 gene products functional assignments could be made. Twenty-six and 28 products are similar to proteins of K139 and the prophage of *V. furnissii* NCTC 11218, respectively, with average identity values of 62.5 and 71.4% (Table S1). However, while the left part of the PV94 genome is more similar to K139, the genes of the right side are much more homologous to the *V. furnissii* prophage (Fig. 2B).

As in these two phages, the PV94 integrase gene is located close to the left genome end. Yet, its product revealed only similarity to the K139 integrase and not to that of the *V. furnissii* prophage. Next to the integrase gene the PV94 genome harbors an immunity region which contains ORFs whose products are related to the prophage repressor CI, lytic repressor Cox and regulatory protein CII of other *Vibrio* phages. However, we did not detect a K139 *glo*-like gene that was suggested to encode a virulence-associated protein [9].

**Figure 3:**
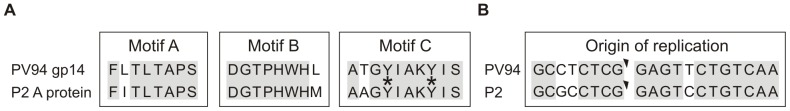
Comparison of the PV94 and P2 replication proteins and origins of replication. (A) Conserved motifs (A, B and C) of the replication proteins. The two tyrosine residues within motif C which are part of the active site of P2 protein A are marked by an asterisk. (B) Alignment of the P2 origin of replication located within gene *A* with the corresponding region of PV94 ORF14. Arrows indicate the cleavage site at the replication origin.

The main replication protein of PV94 is obviously encoded by ORF14. Its product shows significant homology (53 to 66% identity) to replication proteins of other *Vibrio* phages. Interestingly the protein is also related to protein A of P2 (33% identity) known to replicate by rolling circle replication [24]. Three conserved motifs (A, B and C) including two tyrosine residues within motif C which are part of the active site of the P2 protein A [25], also exist in the PV94 ORF14 product (Fig. 3). Moreover, the possible PV94 origin of replication (*ori*) which is nearly identical to the P2 *ori* located in the *A* gene [26] was found within ORF14. Though, a product similar to P2 protein B that is needed for lagging-strand synthesis during lytic replication [27] was not identified in PV94.

**Figure 4:**
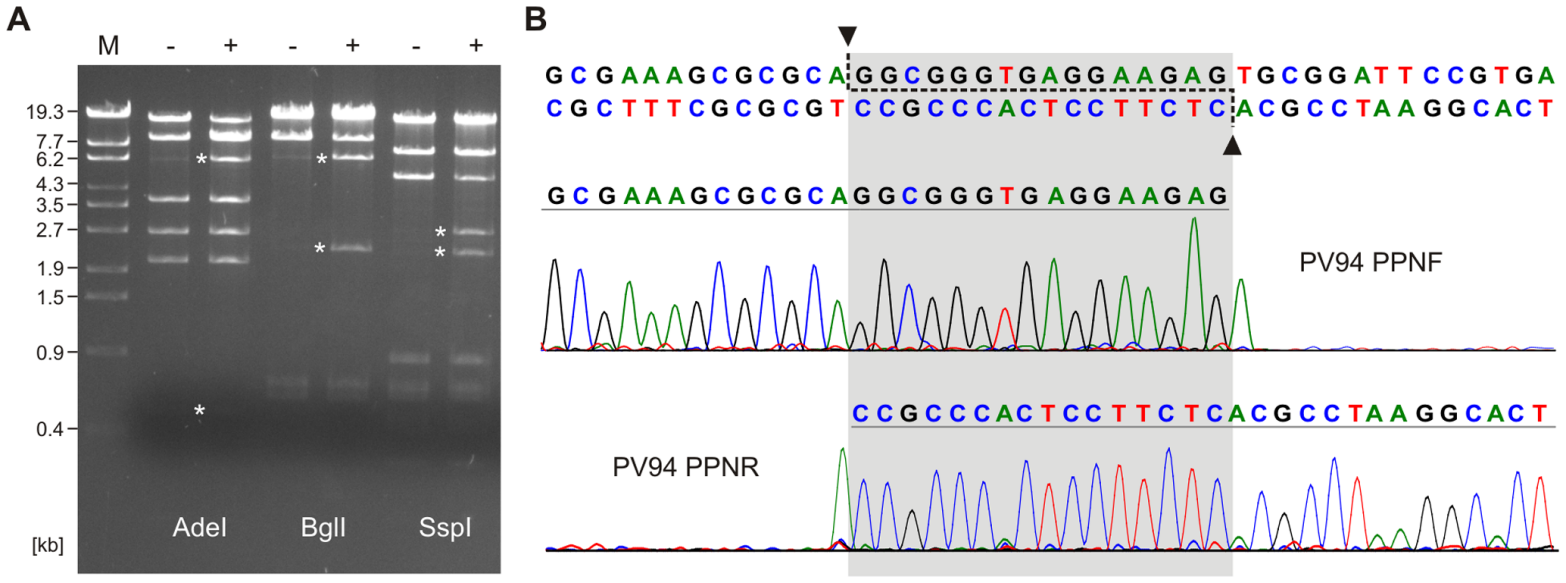
Analysis of the PV94 cohesive ends. (A) AdeI, Bgll and SspI restriction patterns of PV94 phage DNA. Lane M, DNA size marker (λ DNA Eco130I), lanes (−), unheated phage DNA, lanes (+), phage DNA that had been heated before digestion. (B) Determination of protruding nucleotides. Chromatograms of run-off sequencing reactions using phage DNA as template and primers (PPNF and PPNR) binding close to the genomic ends. The 16 bp overhanging sequences are shaded and marked by arrows.

As with the replication proteins the probable PV94 large and small terminase subunits encoded by ORF24 and ORF27, respectively, are very similar (85 and 80% identity) to the respective proteins of K139 and they are also closely related to the terminase subunits of the *V. furnissii* NCTC 11218 prophage. In K139 terminal redundant ends diverging from the *cos*-ends of P2-like phages have been identified [9] even though the large terminase subunits of these phages fall into the same group [28]. We analysed the ends of the PV94 genome by restriction analyses and direct sequencing of phage DNA. As shown in Fig. 4A restriction fragments appeared in heated PV94 DNA that were absent or hardly visible in the unheated control digests. Upon treatment of the phage DNA with T4-ligase these additional fragments did not emerge (data not shown). Direct sequencing of phage DNA using primers that bound adjacent to the genomic ends confirmed the presence of 5′-overhangs 16 bp in length (Fig. 4B). This clearly showed that despite the close relationship of their terminases, phage PV94 and K139 possess different genome end structures. Thus, while K139 DNA might circularize after infection by recombination of its terminally redundant ends, in PV94 circularization is obviously accomplished by *cos*.

The PV94 terminase genes are embedded into a cluster of ORFs probably coding for head proteins (Fig. 2B). A portal protein (ORF23), a capsid scaffolding protein (ORF25), a major capsid protein (ORF26) and a head completion protein (ORF28) have been determined (Table S1), of which the major capsid protein has also been identified by MALDI-TOF (data not shown). Yet, it is striking that among the head proteins only the predicted portal protein is very similar (86% identity) to its K139 counterpart, whereas the other head proteins encoded by genes to the right showed only identity values between 30 to 63% (Fig 2B). Furthermore, most PV94 tail proteins whose genes (ORFs 29, 31, 32, 40, 42, 43, 44) are residing downstream from the predicted head assembly genes are only distantly or not related to K139 proteins. While the overall similarity of this part of the PV94 genome to K139 is rather low (exempt from the lysis gene cluster, see below), its similarity to the *V. furnissii* NCTC 11218 prophage is strong (Table S1). None of the identified head and tail proteins is less than 61%, most of them, however, more than 80% identical to the respective proteins of *V. furnissii* NCTC 11218. Unfortunately, there is no information available about the activity of the prophage in this strain. As mentioned above we could not find an indicator strain for PV94. Neither the morphology nor the gene content of the phage gave any clue for a possible defect. We found ORFs for structural tail proteins, a tape measure protein, and tail assembly proteins, not homologous but similar in size as their counterparts in K139. The tape measure protein (ORF40) and a baseplate assembly protein (ORF42) have also been identified by mass spectrometry (data not shown). Though, unlike with K139-related phages the predicted PV94 tail fiber protein did not show a mosaic-like structure supposed to determine the host range of *V. cholerae* phages [21]. Hence, it is likely that PV94 is highly specific with respect to its host or that the phage contains a yet unidentified mutation that prevents its infectiousness. Such a defect would probably not be caused by a mutation or lack of lysis genes as a cluster of ORFs whose products are closely related to the proposed phage lysin, holin and a lytic accessory protein of K139 is embedded in the tail gene region. It is striking that this short stretch of DNA shows significant homology to K139 but not to the *V. furnissii* NCTC 11218 prophage and further demonstrates the mosaic structure of the PV94 genome.

In conclusion this is the first description of a temperate myovirus of *V. vulnificus*. Phage PV94 did not reveal relationship to the *V. vulnificus* phages SSP002 and VvAW1 that have been recently described [12], [13]. Instead it is similar to temperate phages of other *Vibrio* species. The PV94 genome contains several clusters of genes responsible for specific functions, e.g. head and tail assembly, host cell lysis or the genetic switch for the lytic and lysogenic cycle. The modular organization of the PV94 genome and the varying homologies to other phages suggest recombinations to be responsible for this mosaicism. As most of the PV94 structural proteins are much more distantly related to proteins of K139-like phages than e.g. the terminase subunits or the replication protein, it is not surprising that PV94 did not infect *V. cholerae* strains. Why it also did not lyse *V. vulnificus* is still obscure. We checked the tested indicator strains for own prophages which may cause homoimmunity to PV94 but could not isolate any phage from these strains. Notably, we induced some prophages in other *V. vulnificus* strains which showed similar restriction patterns to PV94 (data not shown). Also for these phages to date suitable indicator bacteria have not be identified. So while prophages are obviously widespread in *V. vulnificus*, their host specificity and the role which they play for pathogenicity are still unclear. Even though a *glo*-like gene or other phage-associated virulence genes (e.g. for toxins, proteins that thwart the host defence, tissue invasion factors) have not been identified in PV94, the phage may contain genes which trigger the virulence of its host. Since for many PV94 gene products a functional assignment could not be made as yet, it can not be excluded that prophages may modulate the virulence also in *V. vulnificus*.

### Nucleotide sequence accession number

The genome sequence of PV94 is available under Genbank accession number HG803181.

## Supporting Information

Table S1 **ORF analysis of the PV94 genome.**
(XLS)

Table S2 **PV94 transcription terminators.**
(XLS)
